# Benefits of Teledermatology for Geriatric Patients: Population-Based Cross-Sectional Study

**DOI:** 10.2196/16700

**Published:** 2020-04-21

**Authors:** Mara G Bianchi, Andre Santos, Eduardo Cordioli

**Affiliations:** 1 Telemedicine Hospital Israelita Albert Einstein São Paulo Brazil

**Keywords:** access to and use of services, decision making, epidemiology, economics, health care systems and management (telehealth), management, technology, teledermatology, geriatric population

## Abstract

**Background:**

Teledermatology is a health care tool that has been increasingly used around the world, mostly because dermatology has an emphasis on visual diagnosis. Many studies have shown that access to specialized care improves using teledermatology, which provides accurate diagnosis and reduces the time taken for treatment, with high patient satisfaction. As the population around the world grows old, there will be even more demand for dermatologists in years to come. It is essential to know which are the most prevalent skin conditions in the primary care population and if they can be addressed through teledermatology.

**Objective:**

Our main goal was to evaluate the proportion of lesions in individuals aged 60 years and older that could be managed using teledermatology in conjunction with primary care physicians. Second, we aimed to assess the most frequent skin lesions, the most common treatments provided to patients, and the distribution and causes of referrals made by the teledermatologists.

**Methods:**

This was a retrospective cohort study from July 2017 to July 2018 in São Paulo, Brazil. We included 6633 individuals aged 60 years and older who presented with 12,770 skin lesions. Teledermatologists had three options to refer patients: (1) to undergo biopsy directly, (2) to an in-person dermatologist visit, and (3) back to the primary care physician with the most probable diagnosis and treatment.

**Results:**

Teledermatology managed 66.66% (8408/12614) of dermatoses with the primary care physician without the need for an in-presence visit; 27.10% (3419/12614) were referred to dermatologists, and 6.24% (787/12614) directly to biopsy. The most frequent diseases were seborrheic keratosis, solar lentigo, onychomycosis, melanocytic nevus, benign neoplasms, actinic keratosis, epidermoid cyst, xerosis, leucoderma, and wart, with significant differences between sexes. Malignant tumors increased with age and were the leading cause for biopsies, while infectious skin conditions and pigmentary disorders decreased. Emollient was the most frequent treatment prescribed, in 31.88% (909/2856) of the cases.

**Conclusions:**

Teledermatology helped to treat 67% of the dermatoses of older individuals, addressing cases of minor complexity quickly and conveniently together with the primary care physician, thus optimizing dermatological appointments for the most severe, surgical, or complex diseases. Teledermatology does not aim to replace a face-to-face visit with the dermatologist; however, it might help to democratize dermatological treatment access for patients and decrease health care expenses.

## Introduction

### Background

Teledermatology is a health care tool that has been increasingly used around the world, mostly because dermatology has an emphasis on visual diagnosis. Studies from all over the world have been performed on this subspecialty [1]. Real-time or store-and-forward are the most common types of delivering images. In store-and-forward teledermatology, patient data and images are collected and sent to a dermatologist to analyze at a later time. In real-time teledermatology, patients and physicians exchange data and images from separate locations in real time [2]. In hybrid teledermatology, both types of images and data are used together. Many studies have shown that access to specialized care is improved by using store-and-forward teledermatology, which provides accurate diagnosis and reduces the time taken for treatment, with high patient satisfaction [3]. A metanalysis with 21 studies showed that the diagnostic accuracy (defined as agreement with histopathology for excised lesions or clinical diagnosis for nonexcised lesions) is still higher for the determination of skin cancer in face-to-face dermatologist, ranging from 67% to 85% depending on the study, than teledermatology, which varied from 51% to 85%. However, some studies do report higher accuracy of teledermatology diagnosis [1].

Typical skin conditions such as mild atopic dermatitis, acne, fungal infections, xerosis, and others are common diseases that may be manageable within the primary care attention service. However, this is not often observed because health professionals are not well trained in diagnosing or triaging skin diseases. The lack of well-trained primary care physicians can lead to unnecessary referral of patients to dermatologists. When there are not enough dermatologists to meet demand, appointments can be filled by patients who do not need specialist care limiting availability of visits for those who do need them.

The city of São Paulo has nearly 12 million inhabitants [4], and 58% of them depend exclusively on the public health care system [5]. The public municipal health care system provides most primary care services. By July 2017, 57,832 individuals were waiting for an appointment with a dermatologist, which could take up to 1 year to occur. As the population around the world grows old, there will be even more demand for dermatologists in years to come, as many skin conditions appear or worsen in older patients. It is essential to know which are the most prevalent skin conditions in the primary care population. If they can be addressed through teledermatology, we may be able to optimize the public health system to manage individuals aged 60 years and older properly. This study had the advantage of including a large number of individuals with many types of skin disease. In contrast, most teledermatology articles focus on one disease (melanoma) or a class of diseases (malignant tumors) [6-8].

### Rationale of the Teledermatology Project

Due to this high unmet demand, the municipal health department established a teledermatology project in partnership with Hospital Israelita Albert Einstein, a large private hospital in the city. The aim was to assist patients in primary care, avoiding unnecessary consultations with dermatologists and accelerating in-presence visits and biopsies for those who have more complex, surgical, or even lethal conditions.

### Objectives of the Study

The primary goal was to determine the proportion of dermatosis in the older population (aged 60 years and older) that could be managed in primary care through teledermatology, but the project included patients of all ages. Second, we assessed the distribution of referrals and frequency (according to sex and age), treatment, and causes of the most common skin diseases for patients assisted in the project. Both objectives have been achieved in this study.

## Methods

### Design

This study was approved by the Hospital Israelita Albert Einstein and Municipal Ethics Committees (CAAE: 97126618.6.3001.0086), and it is in accordance with ethical standards on human experimentation and the Declaration of Helsinki. Data were fully anonymized before being accessed, and the institutional review board waived the requirement for informed consent.

As this teledermatology project included a large number of individuals, it was divided into topics of interest to better analyze the subpopulations. The study design and method are similar to ones that have been previously published [9,10]. In summary, it was a retrospective cohort conducted in the city of Sao Paulo, where 10,545 individuals aged 60 years and older were waiting for an appointment with a dermatologist in July 2017. The municipal health department in conjunction with Hospital Israelita Albert Einstein developed a platform and mobile app to be used by health technicians. Photographs were taken with a digital camera and uploaded along with a short clinical history and patient data. The standard protocol for taking pictures was one photo with enough distance to include the entire part of the body in question (face, arm, leg, trunk), a second one in close-up, around 15 cm away from the lesion, and a third one in a lateral view to capture the volume of the lesion. All data were collected and uploaded to a platform accessed only by dermatologists recruited for this project using a secured online process.

Patients on the waiting list for dermatological assistance were phoned by the public health care service and scheduled to go to an appointment in one of three public city hospitals participating in the project. From July 2017 to July 2018, 13 teledermatologists worked to triage patients, first deciding whether the photographs of the lesions and essential clinical history were satisfactory for diagnostic purposes. If not, they would mark the “bad photo” box on the platform and refer the patient for a face-to-face appointment with a dermatologist. If the photos and essential clinical information were of good quality, the triage dermatologists would formulate the most probable diagnostic hypothesis and choose among three referral options for each lesion assessed: (1) directly to biopsy (after which the patient would return to an in-presence dermatologist appointment with the result); (2) to a dermatologist consultation; or (3) back to the primary care physician with the most probable diagnosis and recommended treatment or guidance on how to proceed with the investigation or management of the lesion. If the same patient had more than one lesion with different referrals, a biopsy referral would prevail over dermatologist referral, which would prevail over a back to primary care physician referral. All patients who attended the project were included in this study.

To better analyze the population of interest, patients aged 60 years and older, we divided them into four categories: 60 to 69 years, 70 to 79 years, 80 to 89 years, and 90 years and older. Only dermatologists certified by the Brazilian Board of Dermatology participated in the project to decrease the chance of diagnostic error by teledermatology. In our municipal basic health unit, a direct exam to search for fungal infection is not done. For this reason, suspicious cases of superficial fungal infections received antifungal treatment as a therapeutic test and were reevaluated afterward. If deep fungal infections were suspected, the teledermatologist would refer the patient to biopsy or a dermatologist and not treat the patient before confirmation of the pathogen.

### Statistical Analysis

Missing data were reported when the patient had the photographs taken. If for any reason, such as problems with the platform, the photographs did not make their way to the teledermatologists or the reports made by the teledermatologists were not uploaded to the platform (this was more frequent at the beginning of the project, mostly for technical reasons), those cases were referred to face-to-face dermatologists. All calculations and frequency analyses were done using only the available teledermatologist reports, meaning that missing data and bad photos were not included. For differences between groups (sex), a statistical calculation was done using a 2-tail chi-square with Yates correction test using Prism 6.0 (GraphPad Software). *P*<.05 was considered significant.

## Results

From a population of 10,545 individuals aged 60 years and older waiting for dermatologist consultations, 6633 patients participated in this project (62.90%); 6320 referrals were made, and 313 participants were lost due to technical problems (4.7%). Multimedia Appendix 1 shows the patient demographic data. There were more females waiting for consultation with a dermatologist across all the ages studied. As the age increased, the percentage of patients who responded to the phone call and participated in the project decreased slightly: 65.14% (4276/6564) at age 60 to 69 years and 55.23% (525/1004) at age 80 years and older. A total of 2.23% (148/6633) of patients were referred to a dermatologist due to the poor quality of photographs.

The total number of photographed lesions was 13,432, and 12,614 diagnoses were made. The mean number of photographed lesions per person was 2. Bleeding was present in only 8.09% (982/12,138) of the lesions, but pruritus was reported in 40.62% (4930/12,138) of them, being more frequent in lesions on individuals aged 90 years and older. Photographs with poor quality were calculated at 1.22% (156/12,770; Multimedia Appendix 1), and patient records lost due to technical problems was calculated at 4.9%.

A total of 49.81% (3148/6320) of patients were referred back to their primary physicians, 42.10% (2661/6320) to consultation with an in-presence dermatologist, and 8.09% (511/6320) directly to biopsy; 66.66% (8408/12,614) of all lesions were referred back to the primary care physician, 27.10% (3419/12,614) to the in-presence dermatologist, and 6.24% (787/12,614) directly to biopsy. The mean waiting time for a face-to-face dermatologist was 6.7 months before the project, which dropped to 1.5 months during the project (reduction of 78%).

**Figure 1:**
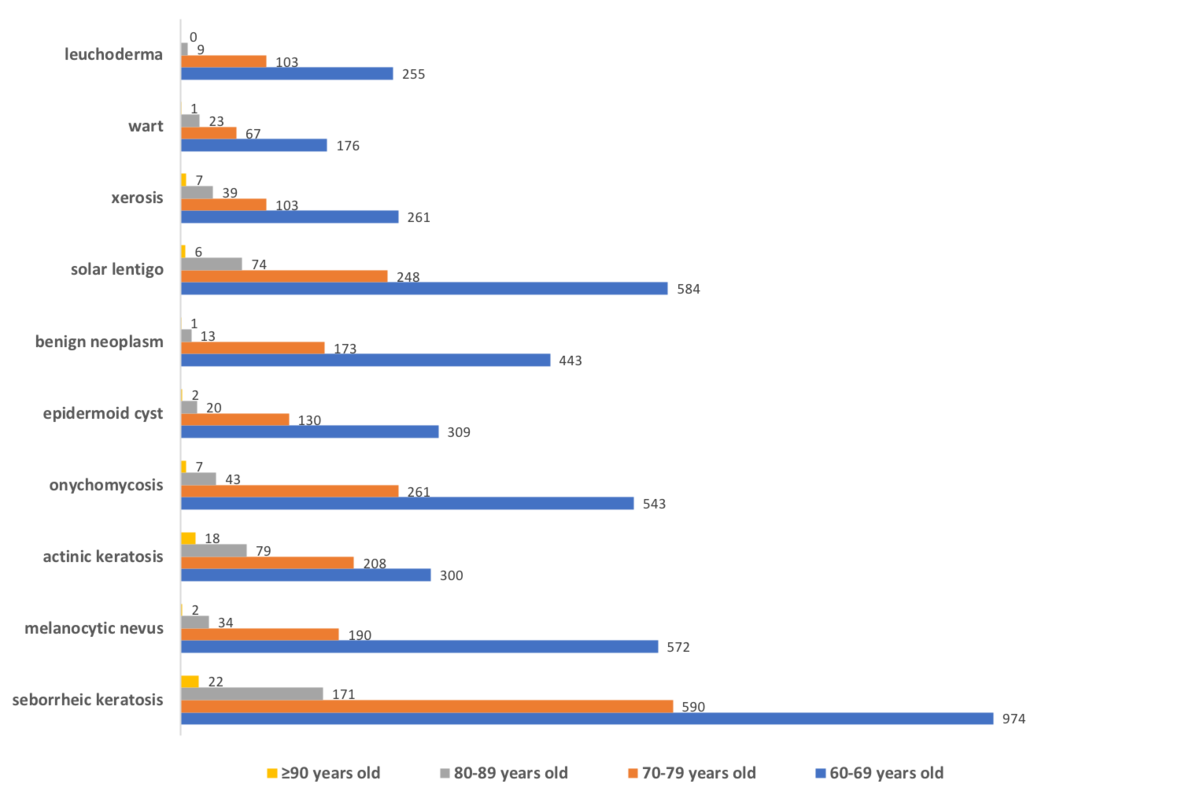
Most frequent diseases (n) in patients aged 60 years and older who participated in the teledermatology project according to sex from July 2017 to July 2018, in São Paulo, Brazil.

The most common causes of consultation for individuals aged 60 years and older are shown in Figure 1. Seborrheic keratosis was the leading cause for teletriage consultation, totaling 13.93% (1757/12,614) of all complaints. Other benign tumors, such as melanocytic nevus, benign neoplasms, and epidermoid cysts, accounted for 14.96% (1889/12,614). Pigmentary disorders (solar lentigo and leucoderma) were also very frequent, accounting for 10.38% (1309/12,614) of the lesions. Actinic keratosis alone was responsible for 4.80% (605/12,614) of the diagnoses, infectious diseases such as onychomycosis and warts were fairly common (6.77% [854/12,614] and 2.12% [267/12,614] of the cases, respectively), and xerosis was responsible for 3.25% (410/12,614) of all complaints. There were significant differences in the frequency of lesions according to sex. Epidermoid cyst (*P*<.001), wart (*P*=.04), and actinic keratosis (*P*=.008) were more frequent in men. Solar lentigo (*P*<.001), onychomycosis (*P*=.02), leucoderma (*P*=.01), and melanocytic nevus (*P*<.001) were statistically more frequently diagnosed in women. Seborrheic keratosis, xerosis, and benign neoplasms showed no difference in frequency between the sexes.

**Figure 2:**
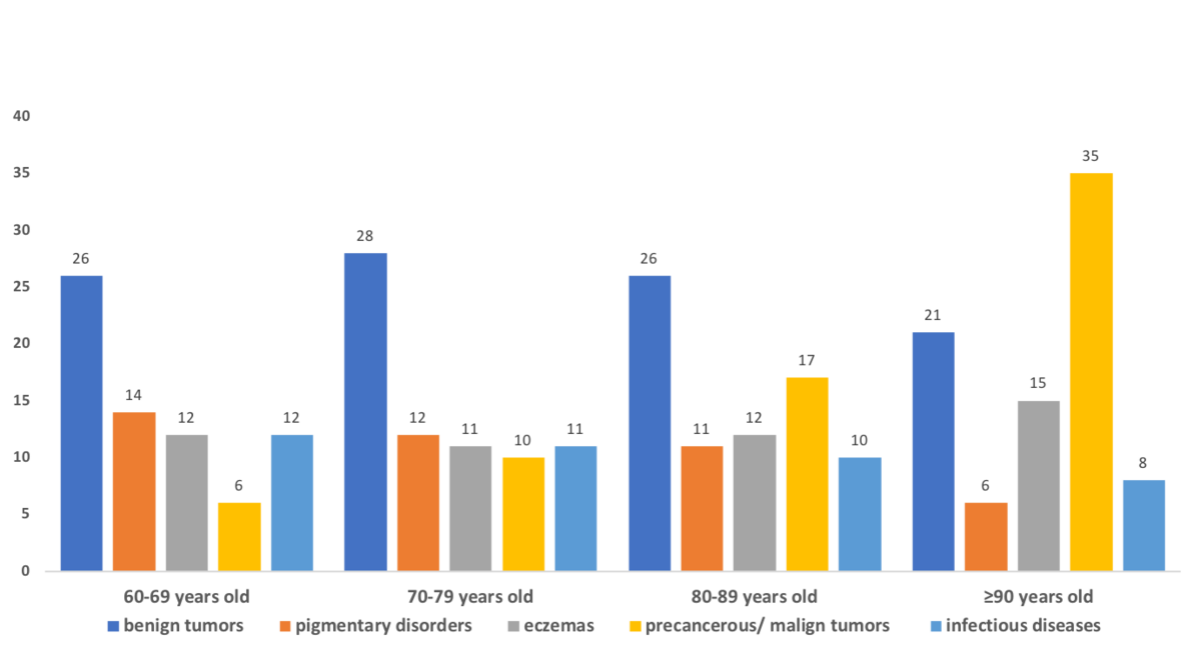
Most frequent group of diseases (%) in patients aged 60 years and older who participated in the teledermatology project by group age from July 2017 to July 2018 in São Paulo, Brazil.

We assessed the most common diseases according to age and divided them into disease groups (Multimedia Appendix 2). Benign tumors were the most frequent complaint for patients up to age 89 years, accounting for about 28.17% (3551/12,614) of the cases. Pigmentary disorders were the second cause for consultations, but this slightly decreased with age. Eczemas were the third cause, very steady in percentage over the years (mean frequency of 12.28% [1549/12,614]. Precancerous and malignant tumors were the fourth cause of visits in individuals aged from 60 to 69 years. Still, the rate increased a lot with age, reaching 34.65% (44/127), and was the leading cause of teletriage consultations in individuals aged 90 years and older. Infectious skin diseases occupied fifth place at age 60 to 69 years old, and the rate gradually decreased with age (Figure 2).

**Figure 3:**
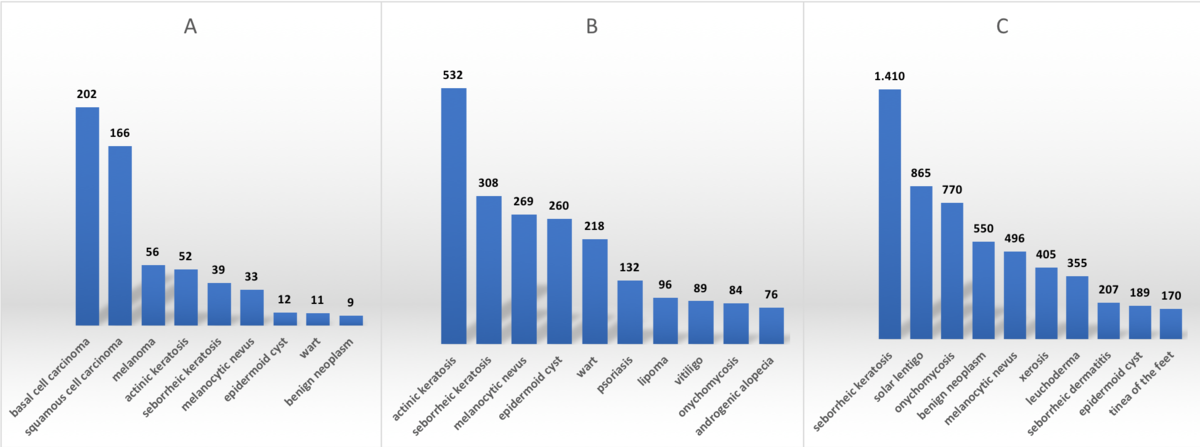
Most frequent lesions (n) sent to biopsy, dermatologists, and back to physician in patients aged 60 years and older who participated in the teledermatology project from July 2017 to July 2018 in São Paulo, Brazil.

The most frequent lesions referred to biopsy, dermatologists, and back to the primary care physician are shown in Figure 3. Biopsy was indicated in 6.24% (787/12,614) of all lesions. Malignant tumors were the most frequent cause: basal cell carcinoma, squamous cell carcinoma, and melanoma being the top 3 indications, in 83.79% (202/244), 90.71% (166/183), and 76.68% (56/74) of the cases with these diseases, respectively. Only 8.60% (52/605) of actinic keratosis cases were sent to biopsy. Seborrheic keratosis, melanocytic nevus, epidermoid cyst, warts, and benign neoplasms such as soft fibroma and acrochordon accounted for 65 cases that were referred to biopsy, representing less than 0.27% (104/3913) of all lesions with this diagnosis.

Referrals to dermatologists occurred in 27.10% (3419/12,614) of the lesions. Actinic keratosis was the lesion which most frequently sent the patient to an in-presence appointment (in 87.93% [532/605] of the times), followed by warts (218/267, 81.65%), epidermoid cyst (260/461, 56,40.%), lipomas (96/245, 39.18%), melanocytic nevus (269/798, 33.71%), and seborrheic keratosis (308/1757, 17.53%). Chronic skin diseases that frequently require follow-ups, such as psoriasis, vitiligo, onychomycosis, and androgenic alopecia, were also in the top 10 causes for referrals to dermatologists. Frequency of referral was 60.27% (132/219), 74.17% (89/120), 9.84.% (84/854), and 32.34% (76/235), respectively.

Teledermatologists sent the patients back to their primary care physicians in 66.71% (8408/12614) of cases. Seborrheic keratosis could be treated at primary care in 80.25% (1410/1757) of cases, solar lentigo in 94.85% (865/912), benign neoplasms in 87.30% (550/630), leuchoderma in 96.73% (355/367), melanocytic nevus in 62.16% (496/798), and epidermoid cyst in 41.00% (189/461). Onychomycosis was managed by teledermatology in 90.16% (770/854) of cases, xerosis in 98.78% (405/410), seborrheic dermatitis in 90.79% (207/228), and tinea on the feet in 93.92% (170/181) of cases.

We also searched for the most frequent treatments prescribed by teledermatologists. Emollients were the most common prescription, in 31.88% (909/2856) of all cases. The other top classes of drugs prescribed were as follows: topical antifungal in 29.52% (843/2856), sunscreen in 27.87% (796/2856), topical corticosteroids (low and high potency) in 24.40% (697/2856), oral antifungals in 7.18% (205/2856), and hydroquinone in 1.33% (38/2856) of cases.

## Discussion

### Principal Findings

The vast majority of the dermatoses assessed did not require an in-presence evaluation by a dermatologist (73%), suggesting that most skin conditions in our primary care setting are of low complexity and, therefore, can be addressed appropriately without referral to more specialized centers. This finding also reinforces the feasibility and importance of teledermatology in this context, helping primary care physicians to manage such conditions and optimizing patient access to care for more severe, surgical or complex illnesses, which do require a dermatologist, especially in the public system. The reduction in the mean waiting time for a face-to-face dermatologist during the project is a great advantage, especially considering that many of these cases could be skin cancers. During the study, the patient was always treated by a physician, which gave us more confidence that potentially severe, life-threatening, or rare diseases were not being overlooked. This work shows that teledermatology is a well-defined, structured, scalable process with standardized collection and fairness of care, which might promote the democratization of access to dermatology for underprivileged patients.

Most of the cases referred for biopsy were patients with malignant lesions, especially basal cell and squamous cell carcinomas. Melanoma, although rarer, was the third cause of biopsy referrals, which is of great concern, since it can lead to death if not promptly diagnosed and treated. The fact that biopsy was indicated in 74% and not 100% of the melanoma cases, as expected, could reflect the teledermatologists’ fear about the type of biopsy that would be performed in those cases: incisional versus excisional. They probably sent the melanoma patients to the dermatologist aiming to ensure the indication of excisional biopsy, which is the gold standard treatment for this disease [11]. Only a fraction of patients with common benign tumors were sent to biopsy (less than 0.5%).

Referrals to dermatologists were made mostly for treatment of premalignant lesions (actinic keratosis) and warts, lesions that usually require procedures such as cryotherapy with liquid nitrogen, excision, curettage, and electrocoagulation. A small portion of patients with benign tumors, such as melanocytic nevi and seborrheic keratosis, were sent to an in-presence dermatologist consultation. Still, these numbers could be even smaller if access to dermoscopic images were granted [10]. Dermoscopy devices permit a 20× augmentation of skin lesions and may help in the diagnosis of pigmented lesions and skin cancer, especially in incipient lesions. Vitiligo, psoriasis, onychomycosis, especially if treated with oral antifungal medicines, and androgenic alopecia cases were also sent to dermatologists, mostly because they are chronic conditions that require follow-ups.

Regarding the frequency of lesions, seborrheic keratosis was the most common complaint found in the study. It is one of the most common skin tumors, but due to its benign nature, treatment is not mandatory. The second condition in frequency was solar lentigo, a benign lesion associated with aging, constituting a general aesthetic and social concern. It appears mostly on chronically sun-exposed surfaces (face and scalp, dorsum of the hands, neckline, and upper back) and is frequently present among the elderly population. Estimates are that more than 90% of white patients aged older than 50 years are affected [12]. Onychomycosis was the third most frequent cause of complaints. Its worldwide prevalence is estimated at 5.5% of the population, and it is more common in older individuals [13]. Nevus was the fourth most frequent concern, about which there are very little data in the older population. Benign neoplasms, such as acrochordon and soft fibromas, were very common complaints in our research. Actinic keratosis was next in prevalence, corresponding to 5% of all lesions. Actinic keratoses are common lesions representing a step in the development of squamous cell carcinoma. Induced by ultraviolet radiation, actinic keratoses increase in number with age and were found to be the most common reason to visit a dermatologist [14]. Epidermoid cysts are one of the common benign subcutaneous tumors, and males are more affected than females [15]. Xerosis (dry skin) was the primary cause of consultation in 3% of our cases, but one study in nursing homes with older persons (mean age 75 years) observed a prevalence of 56% [16]. Classifying diseases in groups and assessing their prevalence according to age, we observed that benign tumors and eczematous diseases showed stable rates. Precancerous and malignant tumors increased 6-fold with age, from age 60 years to older than 90 years, becoming the most prevalent group disease in the oldest population; our findings were similar to study in Turkey [17].

Regarding treatment, a list of drugs available to the population studied is found in Multimedia Appendix 3. All physicians involved in the project were encouraged, as much as possible, to use only medications from this list, since they were available free for patients, who would be unable to buy most of them otherwise. The results showed the importance of emollients in skin conditions, prescribed in one-third of the cases. Topical antifungals were the second most prescribed medication due to the high rate of fungal diseases. Sunscreen was third, mainly to prevent new cases of actinic keratosis and malignant tumors. Low-potency and high-potency corticosteroids also played an important role, rounding out the top 5 treatments list.

### Limitations

One significant limitation of our work is the chance of error and bias in teledermatology diagnosis. However, many articles have attested to a high agreement rate between teledermatology and in-presence dermatology diagnosis [6,18]. The fact that teledermatologists receive multiples photographs of parts of the body and head but are not able to examine the body as a whole makes the diagnosis more challenging. Also, some critical impressions that would help to corroborate the diagnosis, such as feeling the texture of the skin, or proceeding with easy tests (pe vitrocompression) cannot be done. However, in a previous study we have shown that the inability to palpate the lesion is not a major issue for teledermatologists, who became much more confident in teledermatology after working with it [10]. As this teledermatology project was designed to be a triage program, patient follow-ups with the teledermatologists were not included in the study, making it impossible to evaluate the efficacy of the dispensed treatment and gather more information about further referrals made to these patients.

### Comparison With Prior Work

Only one study including 500 patients analyzed the proportion of in-person consultations that could be avoided using teledermatology and showed that in 73% of patients, no referral to an in-person dermatologist was needed [19].

### Conclusions

The current teledermatology initiative in São Paulo managed most of the patients’ dermatoses without the need for an in-presence appointment with a dermatologist, keeping them within the primary care setting. The mean waiting time reduction for an in-presence visit was a definite benefit for the population who presented with more complex diseases. Although it does not aim to replace a face-to-face visit with a dermatologist, teledermatology proved to be an efficient tool for triage and management of less complicated skin conditions quickly and conveniently, and, in our project, it has promoted the democratization of access to dermatology for underprivileged patients. Future works should focus on the cost effectiveness of the project, on the accuracy of teledermatology in inflammatory and tumoral dermatoses, and on the efficacy of the management proposed by teledermatologists to general physicians.

The frequency of skin diseases differed according to age and sex. Recognizing the prevalence and distribution of the most common diseases affecting individuals aged 60 years and older is crucial to address social and health policies that could orient treatment or prevention through popular campaigns and advertisements.

## Appendix

Multimedia Appendix 1 Individuals aged 60 years and older waiting for a dermatologist consultation who participated or not in the teledermatology project and the characteristics of their lesions according to age and sex from July 2017 to July 2018 in São Paulo, Brazil.

Multimedia Appendix 2 List of most common diseases included in the disease groups.

Multimedia Appendix 3 Table of medications available for the teledermatologists from the municipal health system in São Paulo, Brazil, from July 2017 to July 2018.
