# Supplementary material for: Benefits of Teledermatology for Geriatric Patients: Population-Based Cross-Sectional Study
Source: J Med Internet Res. 2020 Apr 21;22(4):e16700. doi: 10.2196/16700 (PMC7201316; doi:10.2196/16700)
Supplement: Multimedia Appendix 1 [file jmir_v22i4e16700_app1.docx]

Multimedia Appendix 1. Individuals aged 60 years and older waiting for a dermatologist consultation, who participated or not in the teledermatology project, and characteristics of their lesions, according to age and sex, from July 2017 to July 2018, in the city of São Paulo, Brazil.

| **PATIENTS (n = 6,633)** | **60–69 years** | | **70–79 years** | | **80–89 years** | | **≥90 years** | |
| --- | --- | --- | --- | --- | --- | --- | --- | --- |
|  | Female | Male | Female | Male | Female | Male | Female | Male |
| Waiting (n) | 4561 | 1973 | 1988 | 1019 | 574 | 303 | 95 | 32 |
| Participating n (%) | 2980 (65) | 1296 (66) | 1181  (59) | 651 (64) | 291  (51) | 166 (55) | 50  (53) | 18  (56) |
| Lesions reported (n) | 6268 | 2401 | 2508 | 1215 | 624 | 289 | 102 | 25 |
| Mean lesions per person | 2.1 | 1.9 | 2.1 | 1.9 | 2.1 | 1.7 | 2.0 | 1.4 |
| Poor quality photo | 75 | 25 | 30 | 9 | 5 | 4 | 0 | 0 |
| To biopsy | 171 | 99 | 92 | 68 | 41 | 21 | 14 | 5 |
| To dermatologist | 1134 | 537 | 473 | 276 | 133 | 77 | 28 | 3 |
| Back to physician | 1534 | 592 | 558 | 285 | 103 | 60 | 7 | 9 |
| **LESIONS**  **(n = 12,770)** |  | | | | | | | |
| Bleeding yes (%) | 392  (6) | 229 (10) | 160  (6) | 113  (9) | 30  (5) | 32  (11) | 18  (18) | 8  (32) |
| Bleeding no (%) | 5288 (84) | 1922 (80) | 2111  (84) | 993 (82) | 514 (82) | 234 (81) | 78  (77) | 16  (64) |
| Pruritus yes (%) | 2192 (35) | 903 (38) | 943 (38) | 457 (39) | 252 (40) | 108 (37) | 57  (56) | 18  (72) |
| Pruritus no (%) | 3488  (56) | 1248 (52) | 1328 (53) | 649 (57) | 292 (47) | 158 (55) | 39  (38) | 6  (24) |
| Poor quality photo (%) | 78 (1) | 27 (2) | 32 (3) | 9 (1) | 5 (1) | 5 (2) | 0 | 0 |
| To biopsy (%) | 253 (4) | 147 (6) | 144 (6) | 113 (9) | 68 (11) | 30 (10) | 27 (26) | 5 (20) |
| To dermatologist (%) | 1419  (23) | 703 (29) | 616 (25) | 351 (29) | 186 (30) | 103 (36) | 37  (36) | 4  (16) |
| Back to the physician (%) | 4217 (67) | 1398 (58) | 1582 (63) | 689 (57) | 329 (53) | 141 (49) | 37  (36) | 15  (60) |
