# Supplementary material for: Benefits of Teledermatology for Geriatric Patients: Population-Based Cross-Sectional Study
Source: J Med Internet Res. 2020 Apr 21;22(4):e16700. doi: 10.2196/16700 (PMC7201316; doi:10.2196/16700)
Supplement: Multimedia Appendix 2 [file jmir_v22i4e16700_app2.docx]

Multimedia Appendix 1. List of most common disease included in the disease groups.

1. Eczemas:

Stasis dermatitis; atopic dermatitis; contact dermatitis; lichen simplex chronicus; dyshidrotic eczema; prurigo nodularis; pruritus; intertrigo; pityriasis alba; photocontact dermatitis; xerosis; miliaria

1. Benign tumors:

Hemangiomas; lymphangiomas; neurofibroma; dermatofibroma; acrochordon, soft fibromas, syringoma; pilomatrixoma; xanthelasma; seborrheic keratosis; keloid; pyogenic granuloma; melanocytic nevus; hidrocstoma; rhinophyma

1. Pre-cancerous/malignant tumors:

Actinic keratosis; actinic cheilitis; basal cell carcinoma; squamous cell carcinoma; melanoma; mycosis fungoides

1. Pigmentary disorders:

Post inflammatory hyperpigmentation; melasma; vitiligo; solar lentigo; solar leuchoderma; café-au-lait macules; cutaneous amyloidosis
