# Supplementary material for: Benefits of Teledermatology for Geriatric Patients: Population-Based Cross-Sectional Study
Source: J Med Internet Res. 2020 Apr 21;22(4):e16700. doi: 10.2196/16700 (PMC7201316; doi:10.2196/16700)
Supplement: Multimedia Appendix 3 [file jmir_v22i4e16700_app3.docx]

Multimedia Appendix 2. Table of medications available for the teledermatologists by the Municipal Health System in the city of São Paulo, Brazil, for July 2017-July 2018.

| Active ingredient | Type of use | Dose |
| --- | --- | --- |
| Acyclovir | Oral | 200 mg |
| Azithromycin | Oral | 500 mg |
| Cephalexin | Oral | 500 mg |
| Ketoconazole | Cream | 2 % |
| Ketoconazole | Shampoo | 2 % |
| Ciprofloxacin | Oral | 500 mg |
| Clindamycin | Oral | 500 mg |
| Dexclhorpheniramine | Oral | 0.4 mg/ml |
| Fluconazole | Oral | 150 mg |
| Hydrocortisone | Cream | 1% |
| Itraconazole | Oral | 100 mg |
| Ivermectin | Oral | 6 mg |
| Loratadine | Oral | 10 mg |
| Loratadine | Oral | 1 mg/ml |
| Neutrogena body care | Lotion |  |
| Nystatin | Oral | 100,000 u/ml |
| Permethrin | Cream | 5% |
| Permethrin | Solution | 1% |
| Prednisone | Oral | 5 and 20 mg |
| Urea | Lotion | 10% |
| Isotretinoin | Oral | 10 and 20 mg |
| Acitretin | Oral | 10 and 25 mg |
| Cyclosporine | Oral | 25, 50 and 100 mg |
| Cyclosporine | Oral | 100 mg/ml |
| Methotrexate | Intramuscular | 25 mg/ml |
| Methotrexate | Oral | 2.5 mg |
| Calcipotriol | ointment | 50 mc/g |
| Clobetasol | Cream | 0.5 mg/g |
| Clobetasol | Solution | 0.5 mg/g |
| Sunscreen SPF 30 | Lotion |  |
| Dexamethasone | Cream | 1 mg/g |
| Ciclopirox olamine | Nail lacquer | 8% |
| Amorolfine | Nail lacquer | 5% |
